# Depression and Somatic Symptoms in a Non-Western Physician

**DOI:** 10.7759/cureus.13043

**Published:** 2021-01-31

**Authors:** Philip Oh, Kai-Wei Lin, John W Norton

**Affiliations:** 1 Medicine, College of Osteopathic Medicine, William Carey University, Hattiesburg, USA; 2 Psychiatry, Mississippi State Hospital, Jackson, USA

**Keywords:** depression, somatic symptoms, case report, dysphagia, depression in a physician

## Abstract

Because of the high prevalence and association of somatic symptoms in depression, a holistic treatment plan that also targets the associated somatic symptoms can be the cornerstone for such patients. In this paper, we present the case of a 53-year-old male physician with depression associated with the somatic symptoms of dysphagia. The initial failure in treating his swallowing defect led to the deterioration in his condition. Moreover, his unique history, complicated by multifactorial life stressors, also raises the attention that there are a variety of presentations of depression.

## Introduction

Depression has always been a difficult topic because of its negative connotation of weakness or submission in our societies. Certain non-Western cultures, like Japan, are known for the appreciation of self-perseverance where there are limitations to the expression of the internal self. Moreover, people carrying more socioeconomic burdens may be less likely to seek help because of those reasons. The high prevalence and association of somatic symptoms in depression raise the importance of holistic treatment strategies [1]. There are also studies stating that depressive people from different cultural backgrounds can present distinct somatic symptoms. A somatic symptom is any bodily sensation that an individual experiences as unpleasant [2]. Somatic symptoms can include headaches, back pain, and gastrointestinal disturbances. The frequency of somatic symptoms can vary depending on the cultural background and socioeconomic status [3-4]. There may also be a correlation between the intensity of a somatic symptom and the magnitude of depression in a patient, leading to a lower quality of life [5-6].

## Case presentation

A 53-year-old Syrian neurologist was admitted to the hospital on September 23, 2020, with dysphagia and appeared discouraged. After first ruling out anatomical and physiological causes of dysphagia, he underwent psychiatric treatment for over 21 days but showed little to no improvement and was losing weight. His dysphagia worsened to the point where he had to utilize a feeding tube and was frequently spitting out saliva. He was previously treated by two speech therapists who tried to incorporate muscle stimulation, but they did not follow-up with him after the treatment. His past treatment also included lithium as the only medication for his symptoms. Unfortunately, he experienced restlessness from the lithium. 

He appeared to be discouraged and presented with some general anxiety. He denied suicidal ideation and he was not melancholic. He identified two previous big stressors as his history of the break-up of two arranged engagements and his residency. He stayed in the United States for 25 years, while the rest of his family stayed in Syria. He is currently not married and has no children. He has also been sending money to his family in Syria.

Swallowing therapy helped him to improve his dysphagia and he was able to move on to a soft food diet. He could eat three meals per day, but he still needed liquid to help him swallow. He initially was on quetiapine, 25 mg daily, and mirtazapine, 15 mg daily, for his depression. He is currently taking only mirtazapine. On October 27, 2020, he showed a more positive affect and he was happy with his progress. He had gained 10 pounds and was able to walk and exercise.

## Discussion

His past treatment with the speech therapists did not resolve his conditions due to a lack of follow-up. Because of the lack of proper screening, he was initially treated as a psychotic patient, and the side effects (extrapyramidal symptoms) from those medications actually made his mood worse. As a result, his dysphagia became more severe and he became more distressed. The previous deterioration of this patient may have been prevented if the physicians had looked into his history and present illness more holistically. While his dysphagia may have been the main contributor to his depression, the dimensions of his significantly stressful life were formulated by family, finance, religion, and ego. The past break-ups of his arranged marriages may have led to feelings of guilt and regret not being able to obtain a family with a wife and kids. His reason for not following through with his arrangements was because he did not find the arranged fiancés’ personalities suitable for him. Moreover, he is the main financial provider for his family back in Syria, which adds more stress upon his career as a foreign neurologist.

There may also be a sense of loneliness that he experiences as a Syrian who lives in an American community. He mentioned that he goes to a mosque in Memphis, Tennessee over the weekends, which is distant from his home in Mississippi. His willingness to travel far to spend time with a familiar community shows a possibility in how he feels distant in his current living situation. Because of his non-Western cultural background and successful career as a neurologist, he may be more reluctant to ask for medical support, especially for mental health; it added another barrier between us, as medical students, and this 30-year-experienced foreign doctor. His professional status, along with his monetary support towards his family, could provide pressure upon him to limit any emotions that show signs of weakness.

## Conclusions

This case study is a great example of how treating somatic symptoms, like dysphagia, can be the cornerstone for psychiatric patients and how factors, such as cultural background and professional status, can influence the presentation of somatic symptoms for depressed patients.
